# PPARα activation directly upregulates thrombomodulin in the diabetic retina

**DOI:** 10.1038/s41598-020-67579-1

**Published:** 2020-07-02

**Authors:** Akira Shiono, Hiroki Sasaki, Reio Sekine, Yohei Abe, Yoshihiro Matsumura, Takeshi Inagaki, Toshiya Tanaka, Tatsuhiko Kodama, Hiroyuki Aburatani, Juro Sakai, Hitoshi Takagi

**Affiliations:** 1 Department of Ophthalmology, St. Marianna University of Medicine, 2-16-1 Sugao, Miyamae-ku, Kawasaki, Kanagawa Japan; 2 Division of Metabolic Medicine, The University of Tokyo, RCAST, 4-6-1 Komaba, Meguro-ku, Tokyo, Japan; 3 Laboratory of Epigenetics and Metabolism, IMCR, Gunma University, 3-39-15 Showa-cho, Maebashi, Gunma Japan; 4 Research Center for Advanced Science and Technology, The University of Tokyo, 4-6-1 Komaba, Meguro-ku, Tokyo, Japan; 5 Genome Science Division, The University of Tokyo, 4-6-1 Komaba, Meguro-ku, Tokyo, Japan; 6 Molecular Physiology and Metabolism Division, Tohoku University Graduate School of Medicine, 2-1 Seiryo-cho, Aoba, Sendai, Miyagi Japan

**Keywords:** RNA, DNA, Retinal diseases, Diabetes, Diabetes complications

## Abstract

Two large clinical studies showed that fenofibrate, a commonly used peroxisome proliferator-activated receptor α (PPARα) agonist, has protective effects against diabetic retinopathy. However, the underlying mechanism has not been clarified. We performed genome-wide analyses of gene expression and PPARα binding sites in vascular endothelial cells treated with the selective PPARα modulator pemafibrate and identified 221 target genes of PPARα including *THBD*, which encodes thrombomodulin (TM). ChIP-qPCR and luciferase reporter analyses showed that PPARα directly regulated *THBD* expression via binding to the promoter. In the rat diabetic retina, treatment with pemafibrate inhibited the expression of inflammatory molecules such as VCAM-1 and MCP1, and these effects were attenuated by intravitreal injection of small interfering RNA targeted to *THBD*. Furthermore, pemafibrate treatment inhibited diabetes-induced vascular leukostasis and leakage through the upregulation of *THBD*. Our results indicate that PPARα activation inhibits inflammatory and vasopermeable responses in the diabetic retina through the upregulation of TM.

## Introduction

Diabetic retinopathy (DR) is the main cause of blindness among working-age adults, and the worldwide prevalence is approximately 35% in patients with diabetes^1^. DR is characterized by the combination of increased vessel permeability and progressive vascular occlusion. However, the molecular mechanisms underlying the pathways associated with DR have not been fully elucidated. Several biochemical mechanisms have been proposed to modulate the pathogenesis of DR through effects on cellular metabolism, signaling, and growth factors^2^. Implicated pathways include the accumulation of sorbitol and advanced glycation end-products, oxidative stress, protein kinase C activation, and upregulation of the renin-angiotensin system and vascular endothelial growth factor (VEGF). Among them, VEGF strongly promotes angiogenesis, a key mediator of the progression of DR. Inhibition of VEGF (e.g., anti-VEGF therapies) was shown to be effective in the management of DR in numerous studies^3–7^. However, some DR patients respond poorly or incompletely to anti-VEGF therapy. Accordingly, there is still substantial unmet medical need in patients with DR.

Peroxisome proliferator-activated receptor α (PPARα) is a ligand-activated transcription factor that regulates lipid metabolism^8^. PPARα is highly expressed in the liver and is also expressed in the retina^9^. Recently, two large, prospective clinical trials have demonstrated that fenofibrate, a canonical synthetic PPARα agonist, has protective effects against DR in type 2 diabetes patients. The Fenofibrate Intervention in Event Lowering in Diabetes (FIELD) Study reported that fenofibrate therapy significantly reduced the cumulative need for laser therapy for DR^10^. The Action Control Cardiovascular Risk in Diabetes (ACCORD) Lipid Study of combination simvastatin and fenofibrate demonstrated a greater reduction in the progression of proliferative DR in type 2 diabetes patients compared with simvastatin alone^11^. Thus, PPARα may be an emerging therapeutic option in DR.

Recently, it has been reported that PPARα is downregulated by microRNA in the retina of diabetic animal models^12^. Previous studies showed that PPARα exerts protective effects against endothelial dysfunction, neovascularization, vasoregression, and vascular hyperpermeability^9,13–15^. Another recent study has also found that important factors in DR such as VEGF and TNFα are downregulated by PPARα activation in the retina of diabetic animal models^16^. However, the mechanisms by which PPARα activation exerts protective effects against DR are not fully understood.

Fenofibrate, which has a long history of clinical use as a lipid-lowering drug, has poor PPAR subtype selectivity^17^. Fenofibrate treatment sometimes results in elevation of the transaminase, homocysteine, and creatine levels in patients. On the other hand, pemafibrate, a novel selective PPARα modulator, has greater PPARα activation potency and higher subtype selectivity than fenofibrate^18–20^. Therefore, pemafibrate may reduce inflammation and angiogenesis more effectively than fenofibrate in DR patients.

Thrombomodulin (TM) is a transmembrane protein expressed on the surface of endothelial cells and is encoded by the *THBD* gene^21^. TM converts thrombin to the anticoagulant form to reduce blood coagulation and inhibits inflammation in blood vessels^22–26^. Recently, recombinant TM has been developed and can potentially be used to treat patients with inflammatory and thrombotic diseases^27,28^.

In the current study, we performed genome-wide analyses of gene expression and PPARα binding sites in vascular endothelial cells treated with pemafibrate and found that PPARα directly regulates the expression of *THBD* in endothelial cells. Furthermore, PPARα activation inhibited retinal inflammation through the upregulation of TM in a rat model of DR. Thus, upregulation of TM by PPARα activation can be a potential therapeutic strategy against DR.

## Results

### Genome-wide analysis of PPARα-targeted genes in vascular endothelial cells

**Figure 1:**
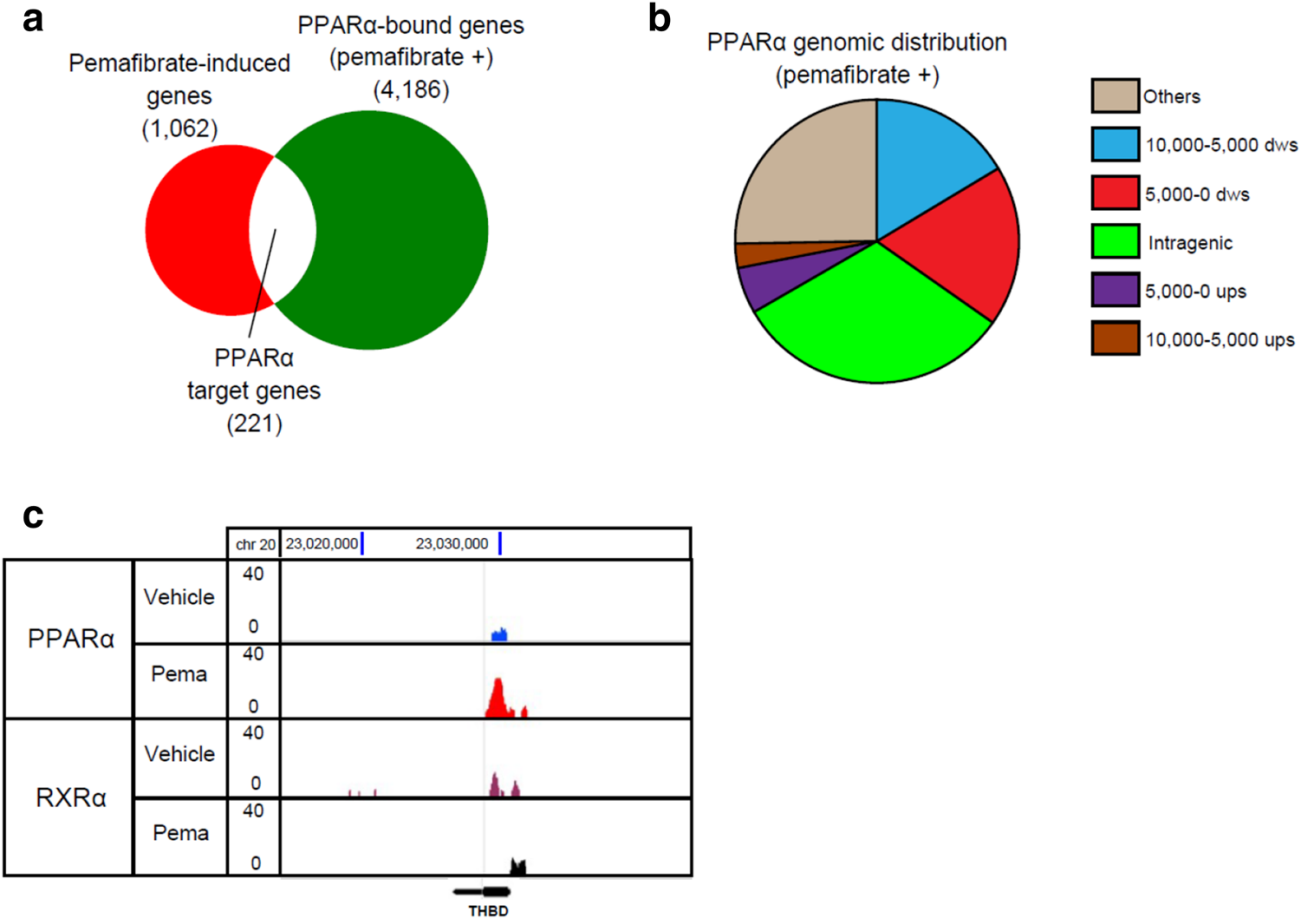
Genome-wide analysis of PPARα binding sites in HUVECs. (**a**) Venn diagram representation of 1,062 pemafibrate-induced genes (≥ 1.5-fold) and 4,186 PPARα-bound genes in HUVECs treated with pemafibrate (10 μM). (**b**) Genome-wide distribution of PPARα binding sites in pemafibrate-treated HUVECs. Ups, upstream; dws, downstream. (**c**) Genome browser representation of PPARα and RXRα binding on *THBD* in HUVECs treated with pemafibrate (pema) or vehicle for 24 h.

To investigate the mechanism of the protective effects against DR by PPARα activation, we performed DNA microarray analysis and ChIP-seq of PPARα in HUVECs treated with pemafibrate. Microarray analysis showed that pemafibrate treatment for 24 h upregulated 1,062 genes (> 1.5-fold, Fig. 1a, pemafibrate-induced genes) and downregulated 477 genes (> 1.5-fold) compared with DMSO treatment (control). Pemafibrate-induced genes included known direct targets of PPARα such as PDK4^18^. The top 50 upregulated and downregulated genes are listed in Tables 1 and 2, respectively.

**Table 1:** List of upregulated genes.

LYVE1	ADAMTS1	LOC100288221	COL4A6	TCERG1L
GALNTL2	LOC727930	CCL23	FAHD2A///FAHD2B///LOC729234	RAB33A
THBD	TCEA3	MNS1	TSPAN7	CDKL1
PCDH17	DNAJC12	AK5	SLC25A30	BMP2
ASNS	EHD3	DPP4	TXNIP	TUBA4A
POSTN	HLX	AIFM3	LOC100509303///LOC100510086	LIFR
PLAT	HLA-DMA	ABHD14B	LOC389834	TFF3
KIAA1466	EDNRB	LIMCH1	C10orf116	MEF2C
SLC1A1	ADAMTS3	HCFC1R1	CASP12	MPZ
PDK4	PLS1	CPS1-IT	CH25H	LOC100270804

**Table 2:** List of downregulated genes.

SELE	VCAM1	IL8	KRT7	LOC100507507
CCL2	C1orf110	CLDN1	DKFZp547G183	PTX3
TGFB2	HSD17B2	GLIPR1	PMEPA1	CYTL1
LYPD1	CXCL1	LRRC17	IL33	COL12A1
DKK1	GJA4	EVI2B	SYNGR3	SPSB1
GUCY1A3	RSPO3	ABCG2	TNKS2	TSPAN2
SEMA3G	GJA5	TOX	F2RL1	TNFRSF12A
CYP4X1	SERPINE2	C8orf48	PLAU	ENC1
DIRAS3	ANXA3	C6orf168	NMU	4-Mar
LOC100506828	PLA2G4C	TMSB15B	ZNF702P	ARSA

In ChIP-seq analysis, peak calling by SICER identified 6,017 genomic regions as significant binding sites of PPARα in pemafibrate-treated HUVECs, and these binding sites were annotated to 4,186 genes (Fig. 1a, PPARα-bound genes). The PPARα genomic binding regions were localized mostly on intergenic regions (49%), and partly on proximal promoters (18%), and intragenic regions (31%) (Fig. 1b). Among PPARα-bound genes, 221 overlapped with pemafibrate-induced genes (Fig. 1a and Table 3). These target genes of PPARα in HUVECs included *PDK4*, *ECH1*, and *ANGPTL4*, which are known metabolic genes induced by peroxisome proliferators^18,29,30^. We also identified *THBD* encoding TM as a target gene of PPARα. A genome browser shot showed pemafibrate-dependent PPARα binding near the transcription start site (TSS) of *THBD* (Fig. 1c).

**Table 3:** List of overlapped genes.

NADK	NR4A2	ZNF827	DYNC1I1	TTLL11	TTC12	NOVA1	CALCOCO2
CTNNBIP1	METAP1D	PDLIM3	CUX1	PTGS1	VWA5A	RALGAPA1	MSI2
MTF1	FRZB	C5orf49	ATXN7L1	RC3H2	CD9	RAD51B	BCAS3
FOXJ3	MYO1B	FAM173B	TFEC	RALGPS1	ATF7IP	ZFP36L1	MED13
LEPR	CFLAR	ANKH	CFTR	ENG	TM7SF3	SPTLC2	ACE
PDE4B	RAPH1	RHOBTB3	GRM8	PTGES	ADAMTS20	FOXN3	MAP2K6
SLC35D1	HDAC4	LOC100289230	CALD1	FNBP1	ANO6	C15orf41	SDK2
AK5	CMTM7	ST8SIA4	RBM33	SURF6	GALNT6	TYRO3	RPTOR
PRKACB	STAB1	KLHL3	UBE3C	EHMT1	ACVR1B	MAPKBP1	SLC16A3
MAGI3	FHIT	ZNF346	CLN8	TUBBP5	ERBB3	TEX9	MEX3C
POU2F1	PTPRG	KIF13A	SLC25A37	IDI2-AS1	9-Mar	GTF2A2	LMAN1
DNM3	CD47	RNF144B	RBPMS	MSRB2	C12orf66	RORA	PIGN
FASLG	LRRC58	BAG6	NRG1	PDSS1	BEST3	DAPK2	CDH19
SEC16B	GOLGB1	TBC1D22B	ASPH	ARMC4	KCNMB4	EMP2	TMX3
DSTYK	KIAA1257	CEP57L1	UBE2W	BICC1	KITLG	EARS2	SBNO2
SLC45A3	ARHGEF26	TRAF3IP2	ZNF704	ANXA2P3	PLXNC1	NFATC3	ANGPTL4
MAPKAPK2	RARRES1	RNF217	RIMS2	SRGN	PARPBP	CDH13	ZNF441
SMYD2	MECOM	PDE7B	SMARCA2	CHST3	MORN3	ZFPM1	LPHN1
HLX	PLD1	MTRF1L	JAK2	RNLS	GOLGA3	SPG7	ZNF493
LYST	ZMAT3	ZDHHC14	C9orf72	CTBP2	FLT1	TRPV2	PPP1R14A
LOC375196	ZCCHC4	WTAP	NFX1	STIM1	LINC00598	SHMT1	ECH1
EML6	PCDH7	AMZ1	PRSS3	CYP2R1	NUFIP1	SYNRG	PCED1A
NPAS2	LIMCH1	SDK1	LOC642236	CPT1A	SLC25A30	ARHGAP23	UBOX5
ST6GAL2	CEP135	GPNMB	C9orf85	ARRB1	PCDH17	EIF1	CDS2
TMEM37	ELOVL6	SKAP2	GABBR2	PCF11	UGGT2	DBF4B	PCSK2
CXCR4	INPP4B	PDK4	ZFP37	ZC3H12C	CLYBL	LOC100506325	TMPRSS15
THBD	WFDC8	ITSN1	UQCC	MRPL39	SGSM3	KLHL22	DYNLRB1
E2F1	BCAS4	DIP2A	TGM2	SYNJ1	DYNLRB1		

We also performed ChIP-seq of retinoid X receptor α (RXRα), which is a heterodimer partner of PPARα, and found RXRα binding near the TSS of *THBD* regardless of pemafibrate treatment (Fig. 1c). Because TM is reported to inhibit inflammation in blood vessels^23,26^, we hypothesize that the upregulation of TM by PPARα activation could inhibit the inflammatory response in the diabetic retina.

### PPARα directly upregulates *THBD* expression

**Figure 2:**
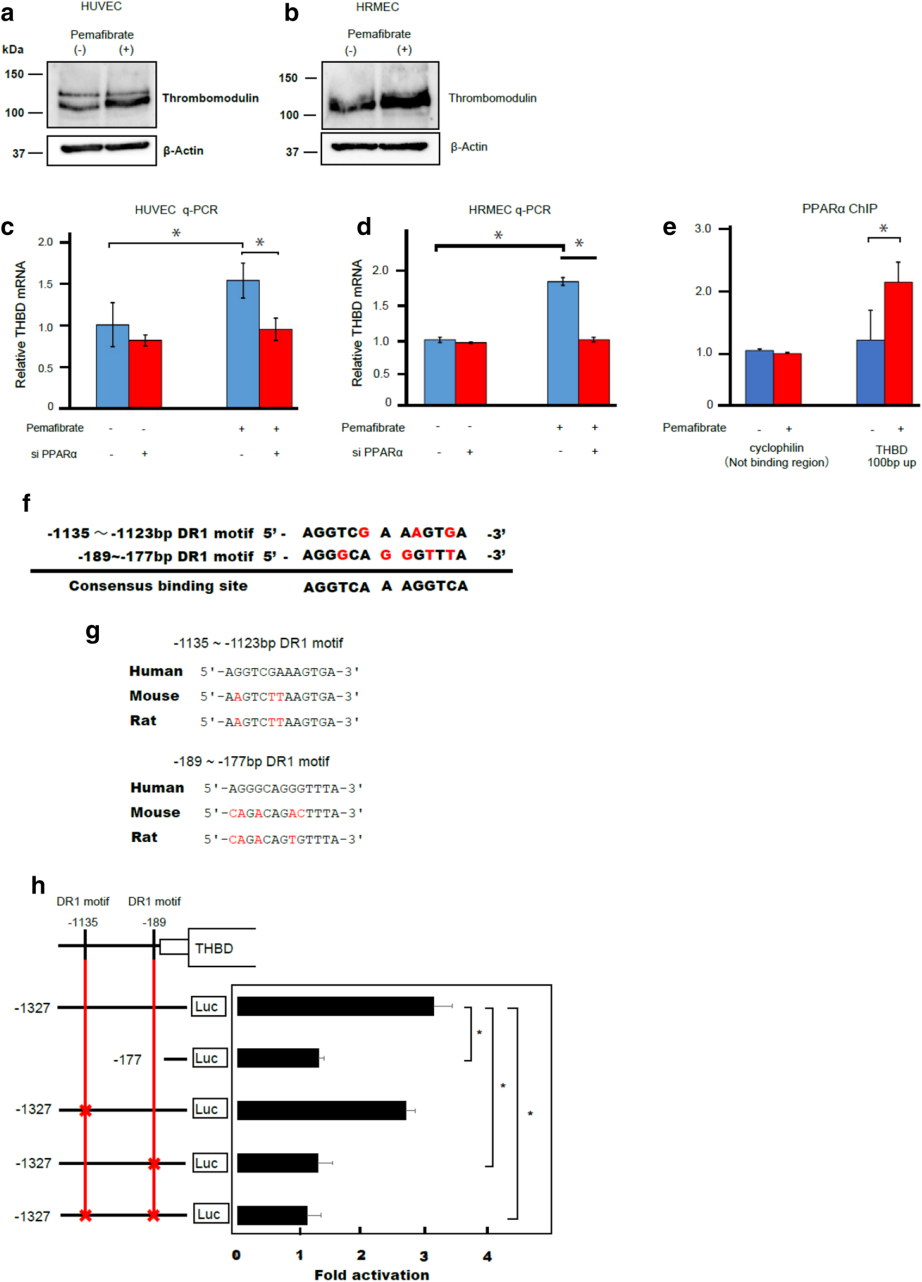
PPARα directly transactivates THBD expression. (**a**,**b**) Immunoblot analysis showing the expression of TM in HUVECs (**a**) and HRMECs (**b**) treated with pemafibrate (10 μM) or vehicle for 24 h. Expression of TM was increased by pemafibrate treatment in both HUVECs and HRMECs. (**c**,**d**) HUVECs (**c**) and HRMECs (**d**) were transfected with siRNA targeted to human PPARα (5 nM) or control siRNA and treated with pemafibrate or vehicle for 24 h. *THBD* mRNA was measured using RT-qPCR. Cyclophilin mRNA was used as the invariant control. Knockdown of PPARα canceled pemafibrate-mediated upregulation of *THBD* in HUVECs and HRMECs. (**e**) PPARα binding on the *THBD* promoter was evaluated by ChIP-qPCR. ChIP signals are presented as fold enrichment. Cyclophilin was used as a negative binding region. PPARα was bound approximately 100 bp upstream from the TSS of *THBD*. (**f**) Alignment of promoter sequences of human *THBD*, mouse *Thbd*, and rat *Thbd* containing two putative DR1 motifs. The DR1 motifs at positions – 1,135 to – 1,123 bp and – 189 to – 177 bp of human *THBD* are conserved in mouse and rat *Thbd*. (**g**) Alignment of the two putative DR1 motifs in the human *THBD* promoter. At the consensus site, the consensus nucleotide found in the PPARα binding sequences is represented by black letters. (**h**) Luciferase reporter analysis using the human *THBD* promoter. HUVECs were transfected with the luciferase reporter containing wild-type *THBD* promoter or indicated deletion and mutations in DR1 motifs together with PPARα and RXRα expression plasmids. After transfection, HUVECs were treated with fenofibric acid for 24 h and subjected to the luciferase reporter assay. The luciferase activity with fenofibric acid was divided by the activity without fenofibric acid and is presented as fold activation. (Results are expressed as mean ± SD of three independent experiments. *p < 0.05. Mann–Whitney *U* test).

A combination of DNA microarray and ChIP-seq analyses of HUVECs identified *THBD* as one of the target genes of PPARα. To confirm this, we performed immunoblot analysis and showed that pemafibrate treatment upregulated TM protein expression in HUVECs as well as HRMECs (Fig. 2a,b). Q-PCR analysis revealed that the upregulation of *THBD* by pemafibrate was blunted when PPARα was knocked down by small interfering RNA (siRNA) targeted to *PPARα* in HUVECs and HRMECs (Fig. 2c,d). Thus, pemafibrate-mediated induction of *THBD* is dependent on PPARα in HUVECs and HRMECs. To determine whether PPARα directly regulates *THBD* expression, we examined the physical and functional interactions of PPARα with *THBD* in ChIP-qPCR and luciferase reporter analysis, respectively. ChIP-qPCR analysis confirmed PPARα binding on the promoter region of *THBD* in HUVECs treated with pemafibrate (Fig. 2e).

PPARα preferentially binds DNA as a heterodimer to the PPAR-responsive element (PPRE), which is composed of two nuclear receptor consensus half-sites of AGGTCA organized as a direct repeat (direct repeat 1 [DR1])^31^. PPARα forms heterodimers with RXRα, and the heterodimers bind the PPRE located in PPARα-regulated genes. Scanning of the promoter sequences of human *THBD* identified two putative DR1 motifs (– 189 bp to 177 bp and – 1,135 bp to – 1,123 bp from the TSS) (Fig. 2f). The former motif is conserved in mouse and rat *Thbd* genes, while the latter is not (Fig. 2g). Accordingly, we generated a reporter plasmid containing the promoter of *THBD* (~ 1.3 kb) and performed luciferase reporter analysis (Fig. 2h). Because pemafibrate affected the control reporter activities (i.e., renilla luciferase and β-gal) (data not shown), we used fenofibric acid as a PPARα agonist in the luciferase reporter assay. HUVECs were transfected with the reporter plasmid together with expression plasmids of PPARα and RXRα and then treated with fenofibric acid to determine PPARα-mediated transactivation. Treatment with fenofibric acid increased the activity of the luciferase reporter containing the *THBD* promoter by threefold. Deletion of two putative DR1 motifs blunted the transactivation by fenofibric acid. Mutation in the DR1 motif at – 1,135 bp from the TSS alone had no effect on fenofibric acid-mediated transactivation, while mutation in the DR1 motif at – 189 bp from the TSS or mutations in both DR1 motifs abolished transactivation. These results indicate that PPARα directly upregulates *THBD* expression in a ligand-dependent manner by binding to the DR1 motif at – 189 bp from the TSS in HUVECs.

### Pemafibrate inhibits TM-dependent retinal inflammation in diabetic rats

**Figure 3:**
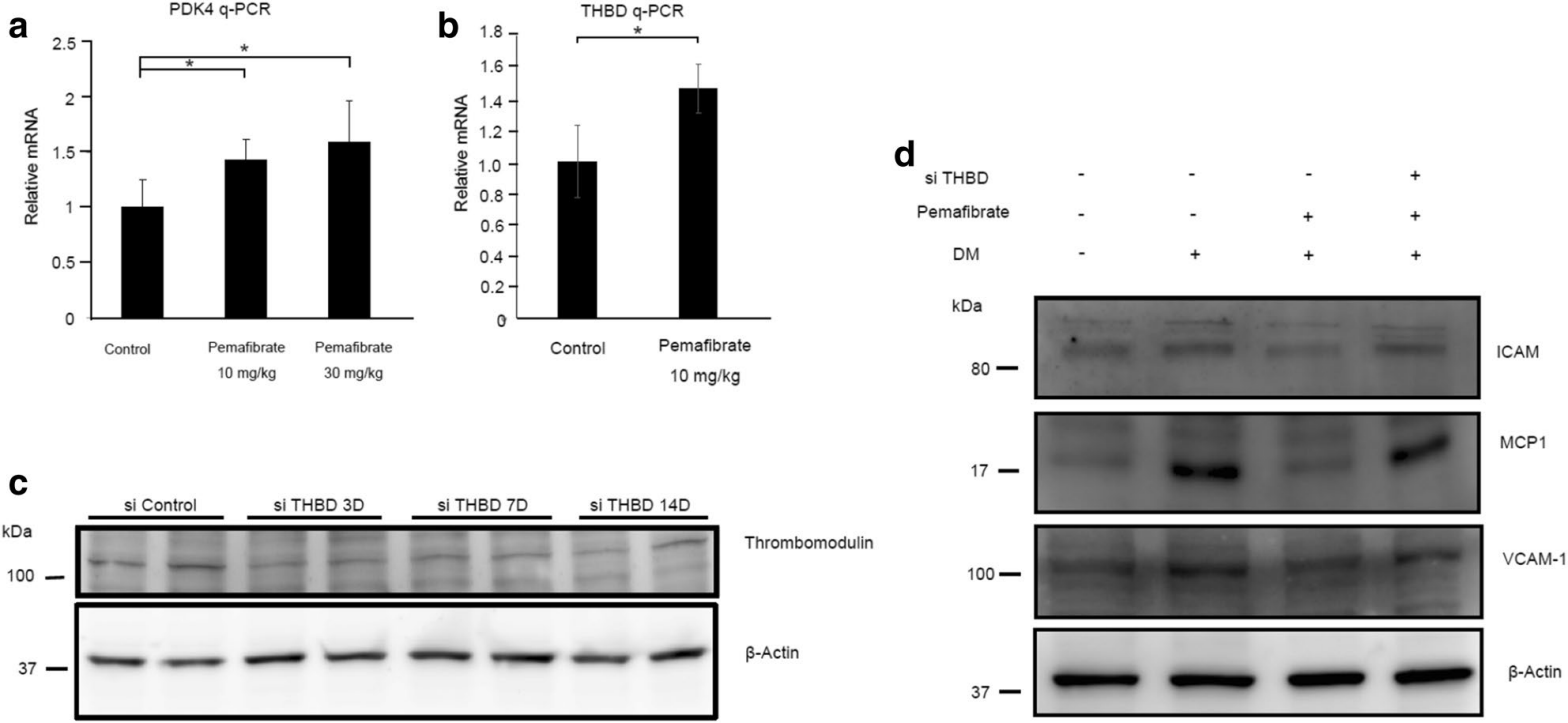
Pemafibrate inhibits expression of inflammatory molecules by upregulating TM in the diabetic retina. (**a**) RT-qPCR analysis showing mRNA expression of *Pdk4* in rat retinas treated with the indicated doses of pemafibrate. *PDK4* is a known PPARα target gene. Pemafibrate treatment significantly upregulated *Pdk4* in the rat retina. (**b**) RT-qPCR showing mRNA expression of *Thbd* in rat retinas treated with pemafibrate. Pemafibrate significantly upregulated *Thbd* in the retina. (**c**) Immunoblot analysis showing expression of TM protein in the retinas of rats intravitreally injected with siRNA targeted to *Thbd* for 3, 7, and 14 days. β-Actin was used as a loading control. The intravitreal injection of siRNA reduced TM protein in the rat retina. (**d**) Immunoblot analysis showing expression of ICAM, MCP2, and VCAM-1 proteins in the retina of control and diabetic rats. β-Actin was used as a loading control. ICAM, MCP2, and VCAM-1 proteins were evaluated in the retinas of diabetic rats compared with control rats. Pemafibrate treatment inhibited elevation of these proteins in the retina of diabetic rats. Knockdown of *Thbd* by siRNA canceled pemafibrate-mediated inhibition of the elevation of these proteins. (Results are expressed as mean ± SD of two independent experiments [**a**,**b**]. *p < 0.05. Mann–Whitney *U* test).

Cell culture experiments showed that PPARα directly upregulated TM, which has antiinflammatory effects. We hypothesized that PPARα activation by pemafibrate could inhibit inflammation through upregulation of TM in the diabetic retina. To examine this, we first administered oral pemafibrate to rats via their feed and showed that pemafibrate (10 mg/kg or 30 mg/kg) significantly increased mRNA expression of PPARα target genes such as *Pdk4* and *Thbd* in the retina (Fig. 3a,b). Next, we set up a TM knockdown system in the rat retina by intravitreal injection of siRNA. Intravitreal injection of 500 pmol of siRNA targeted to *Thbd* for 14 days successfully decreased the protein expression of TM (Fig. 3c). In the STZ-induced diabetic rat model, the protein levels of inflammatory molecules such as ICAM, MCP1, and VCAM-1 in the retina were elevated compared with the retinas of nondiabetic rats (Fig. 3d), as previously reported^32,33^. Oral intake of pemafibrate in STZ-induced diabetic rats markedly inhibited the elevation of inflammatory molecules, indicating that PPARα activation inhibits inflammatory responses in the retinas of diabetic rats. Furthermore, knockdown of TM in diabetic rats attenuated the pemafibrate-mediated inhibition of elevation of inflammatory molecules. These results indicate that the inhibition of PPARα activation by pemafibrate inhibits inflammation through the upregulation of TM in diabetic rat retinas.

### Pemafibrate inhibits TM-dependent retinal vascular leukostasis and leakage

**Figure 4:**
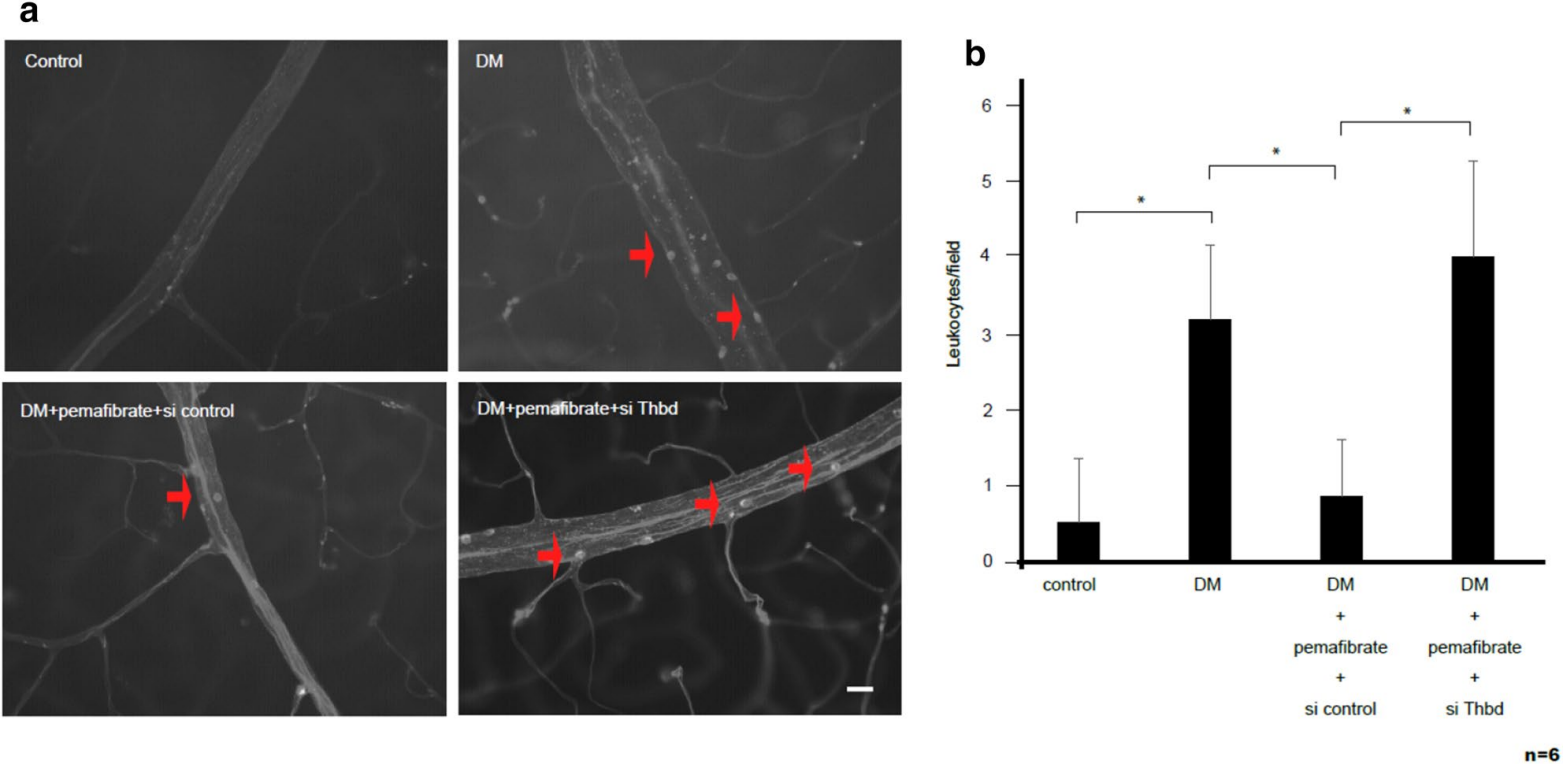
Pemafibrate inhibits retinal vascular leukostasis by upregulating TM. (**a**) Fluorescence microscopy images showing retinal adherent vascular leukocytes in diabetic rats. STZ-induced diabetic rats were treated with pemafibrate or vehicle and intravitreally injected with control siRNA or siRNA targeting *Thbd*. The retinal vascular adherent leukocytes were stained with FITC-conjugated concanavalin-A. Representative images of retinal flat mounts from nondiabetic rats (upper left), untreated diabetic rats (upper right), diabetic rats treated with pemafibrate and control siRNA (lower left), and diabetic rats treated with pemafibrate and si*Thbd* (lower right) are shown. Arrows indicate adherent leukocytes. Scale bar = 50 μm. (**b**) Quantification of adherent leukocytes in retinal images (n = 6 per group). (Results are expressed as mean ± SD. *p < 0.05. Mann–Whitney *U* test).

Vascular leukostasis and leakage increase cumulatively with the progression of DR^34^. To determine whether PPARα activation inhibits vascular leukostasis and leakage in the retina, we examined them in diabetic rats treated with pemafibrate. FITC-labeled adherent leukocytes were not observed in control rats, while they were clearly observed in the retinal vasculature of untreated diabetic rats (Fig. 4a). Pemafibrate treatment significantly reduced the number of leukocytes in the rat retinas (p < 0.05) (Fig. 4a,b), indicating that it inhibits vascular leukostasis in diabetic rats. TM knockdown by siRNA attenuated the pemafibrate-mediated reduction of leukocytes in the retinal vasculature of diabetic rats (p < 0.05) (Fig. 4a,b), while control siRNA treatment did not, indicating that PPARα activation by pemafibrate inhibits vascular leukostasis through TM in the retina of diabetic rats.

**Figure 5:**
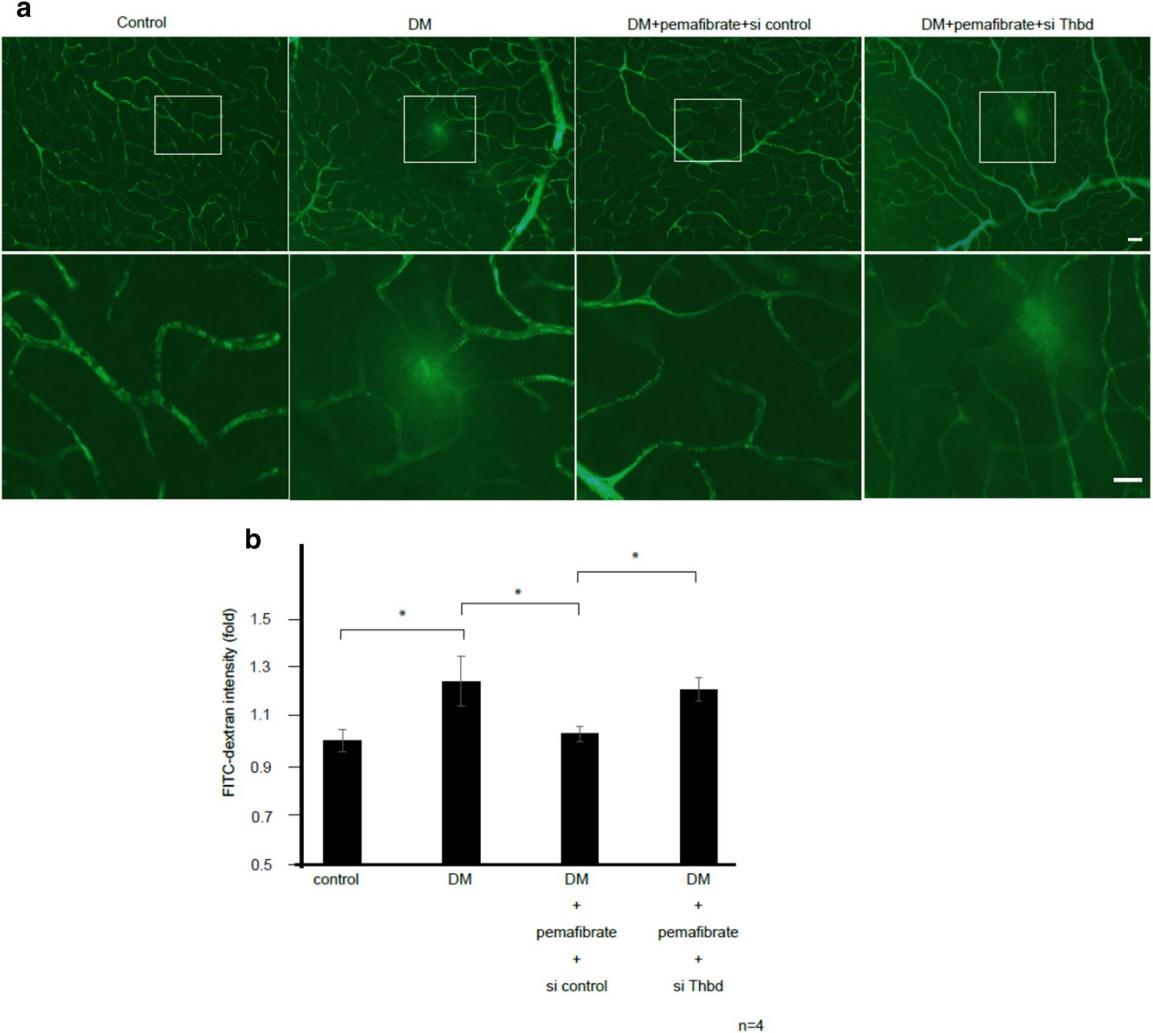
Pemafibrate inhibits retinal vascular leakage by upregulating TM. (**a**) Fluorescent images showing retinal vascular leakage in diabetic rats. STZ-induced diabetic rats were fed pemafibrate or vehicle. STZ-induced diabetic rats treated with pemafibrate or vehicle were then intravitreally injected with control siRNA or siRNA targeting *Thbd*. Vascular leakage was visualized by staining with FITC-dextran. Retinas were examined using fluorescence microscopy. Representative images of retinas from nondiabetic rats, untreated diabetic rats, diabetic rats treated with pemafibrate and control siRNA, and diabetic rats treated with pemafibrate and si*Thbd* are shown. The square areas in the top row are magnified and shown in the bottom row. Scale bar = 100 μm. (**b**) Retinal permeability was quantified by measuring the fluorescence intensity of FITC-dextran in the images on the bottom row (n = 4 per group). (Results are expressed as mean ± SD of three independent experiments. *p < 0.05. Mann–Whitney *U* test).

Next, we examined whether pemafibrate treatment reduces retinal vascular leakage in diabetic rats. The fluorescence intensity of FITC-dextran in the retinal vessels was significantly increased in diabetic rats compared with control rats (p < 0.05), indicating increased vascular leakage in the former (Fig. 5a,b). Pemafibrate treatment significantly reduced the florescence intensity of FITC-dextran in diabetic rats (p < 0.05) (Fig. 5a,b), indicating that PPARα activation by pemafibrate inhibits vascular leakage. In addition, TM knockdown by siRNA attenuated pemafibrate-mediated inhibition of vascular leakage in diabetic rats (p < 0.05) (Fig. 5a,b). These results revealed that PPARα activation by pemafibrate inhibits retinal vascular leukostasis and leakage via TM in diabetic rats.

## Discussion

In the current study, we identified *THBD* encoding TM as one of the direct target genes of PPARα in vascular endothelial cells. PPARα activation by pemafibrate in a rat model of DR upregulated *THBD* and inhibited retinal inflammation and vascular leukostasis and leakage. To the best of our knowledge, this is the first report showing the novel molecular mechanism by which PPARα activation protects against DR.

PPARα is a ligand-activated transcription factor, and its target genes are involved in fatty acid metabolism in tissues with high oxidative rates such as the liver, heart, skeletal muscle, and kidney^8,9^. PPARα is also expressed in other tissues and cells including the intestine, vascular endothelium, and immune cells^15^. Large clinical trials and several reports revealed that PPARα activation by an agonist has protective effects against DR in type 2 diabetes patients^10,11^, although the mechanisms of action of the PPARα agonist were unclear. Because DR is characterized by progressive loss of vascular cells and infiltration of inflammatory cells, we specifically focused on vascular endothelial cells and performed genome-wide analyses of PPARα binding sites and gene expression using ChIP-seq analysis and DNA microarray. Our ChIP-seq analysis identified 6,017 genomic PPARα binding sites, which were assigned to 4,186 proximal genes. Microarray analysis revealed 1,062 genes with expression levels upregulated by pemafibrate treatment in HUVECs.

The majority of PPARα binding sites identified by ChIP-seq were located at intragenic regions or 5′ proximal regions (< 10 kb from the TSS) in HUVECs, consistent with previous ChIP-seq analysis in human hepatoma cells^35^. These results are in line with several studies showing that transcription factor binding sites are generally distributed around the TSS^36,37^. Among 4,186 genes annotated as bound by PPARα, 221 including *THBD* were upregulated by pemafibrate treatment, indicating that these genes are direct PPARα targets in HUVECs. However, pemafibrate treatment did not affect the expression of the majority of genes annotated as bound by PPARα. Some PPARα binding sites may participate in transcriptional regulation of distal genes or in other mechanisms such as noncoding RNA and DNA methylation. It is also possible that our ChIP-seq analysis contained false-positive signals due to the nonspecific binding of antibody. Therefore, the functional relevance of PPARα binding sites needs to be elucidated further.

VEGF has been shown to be one of predominant factors regulating pathological conditions including chronic inflammation and resulting abnormal vasopermeable and angiogenic responses in DR. Our microarray analysis identified 1,062 upregulated and 477 downregulated genes in HUVECs treated with pemafibrate. VEGF mRNA was not altered in the present microarray analysis. Since it is possible that PPARα has transcriptional and nontranscriptional roles in protection against DR, more detailed protein analyses are needed to confirm the direct effects of PPARα activation on VEGF expression. In contrast, the inhibitory effects of PPARα on VEGF expression were clearly demonstrated in in vivo settings. Chen et al. reported the inhibitory effects of fenofibrate on HIF-1 and VEGF expression in the whole retina in a type 1 diabetes rat model and mouse oxygen-induced retinal angiogenesis model^38^. Tomita et al. found that VEGF is suppressed by PPARα activation, which inhibits HIF activity through serum FGF 12 induced and secreted in the liver^39^. Another study showed that PPARα ligands may suppress angiogenesis indirectly by inhibiting tumor cell production of VEGF and FGF2 and by increasing thrombospondin-1^40^. Although we need further detailed protein expression analyses in in vitro studies, these data may indicate that PPARα activation alone does not substantially affect VEGF expression, although it exerts obvious inhibitory effects through cross-reaction with other cytokines and organs. PPARα effects on VEGF-induced intracellular signaling have not been reported and remain to be elucidated.

Among 221 direct PPARα target genes, we focused on *THBD* that encodes TM. TM is an integral membrane protein expressed on the surface of endothelial cells and exerts antiinflammatory effects via several mechanisms^22–26^. TM inhibits inflammation through the activation of protease-activated receptor-1 (PAR-1) in the form of activated protein C (APC)^23,24^. Moreover, TM is a critical cofactor for thrombin-mediated activation of the thrombin-activatable fibrinolysis inhibitor (TAFI)^21^. The proinflammatory mediators are inactivated by TAFI^25^. TM, via thrombin-mediated activation of protein C and TAFI, provides protection against inflammation. Thrombin is an important inflammatory factor in retinal vascular diseases including DR^41^. It was reported that thrombin and prothrombin were increased in the vitreous of patients with proliferative DR compared with nondiabetic individuals^42^. Additionally, it was found that the lectin-like domain of TM sequesters inflammatory factors such as high-mobility group-B1 (HMGB-1) protein and lipopolysaccharide^43,44^. HMGB-1 promotes the process of cell apoptosis by activating the transcription factor nuclear factor-κB after binding to its receptors^45,46^. For these reasons, we hypothesized that PPARα activation inhibited the retinal vascular damage caused by retinal inflammation and apoptosis via the regulation of TM.

In the transcriptional regulation of *THBD*, our studies revealed that PPARα binds to the TSS upstream of *THBD* to activate gene expression. Furthermore, our luciferase reporter assay showed that the DR1 motif at 189 bp from the TSS is responsible for transactivation of the *THBD* promoter by PPARα. Previous studies demonstrated that other transcriptional factors (e.g., Kruppel-like factor 2 and RXRs) also bind to this region to transactivate the *THBD* promoter^47,48^, suggesting the importance of this region for the activity of the *THBD* promoter in vascular endothelial cells.

We used the rat model of DR to show that pemafibrate treatment prevents the upregulation of inflammatory molecules as well as vascular leukostasis and leakage. It was reported that CCL2, VCAM-1, and ICAM-1 are key inflammatory molecules in the diabetic retina which lead to leukocyte adhesion and vascular leakage^32,33^. Inflammatory mediators such as thrombin and HMGB-1 induce these inflammatory molecules^43,45,46^, while TM has inhibitory effects on inflammatory molecules. Therefore, we performed TM knockdown experiments in diabetic rats to determine whether the antiinflammatory effect of pemafibrate is mediated via the action of TM. In vivo experiments demonstrated that the antiinflammatory effect of pemafibrate was canceled by intravitreal injection of Thbd siRNA. These results suggest that the upregulation of TM is one mechanism of the protective effects of PPARα modulators (e.g., pemafibrate) against DR and that a modulator upregulating TM could be a novel therapeutic option for DR treatment. Further studies will be required to elucidate other mechanisms by which PPARα activation protects against DR.

## Methods

### Chemical reagents

Pemafibrate and fenofibric acid were kindly provided by Kowa Co., Ltd. (Nagoya, Japan).

### Cell culture

Human umbilical vein endothelial cells (HUVECs) and human retinal microvascular endothelial cells (HRMECs) were purchased from Lonza (Walkersville, MD, USA) and cultured in EGM-2 MV medium (Lonza) containing 5% FBS at 37 °C in 5% CO_2_.

### siRNA-mediated knockdown of PPARα and THBD

Stealth small interfering RNA (siRNA) (5 nM) targeting human *THBD* mRNA (HSS110719, Invitrogen, Carlsbad, CA, USA) were transfected into HUVECs or HRMECs using RNAiMAX reagent (Invitrogen), and cells were harvested 24 h after transfection. Control siRNA (Med GC) was obtained from Invitrogen. For in vivo experiments, 500 pmol of siRNA targeting rat *Thbd* (RSS331106, Invitrogen) or control siRNA (sc-37007, Santa Cruz Biotechnology, Santa Cruz, CA, USA) was intravitreally injected into streptozotocin (STZ)-induced diabetic rats.

### DNA microarray analysis

HUVECs were treated with 10 μM of pemafibrate or vehicle (DMSO) for 24 h, and total RNA was isolated using Isogen (Fujifilm Wako Chemicals, Osaka, Japan). Preparation of cRNA and hybridization of the probe arrays were performed according to the manufacturer’s instructions (Affymetrix, Santa Clara, CA, USA)^49^. Affymetrix Genechip Human Genome U133 plus 2.0 arrays containing over 54,000 sets were used. Data analysis was performed using GeneSpring GX 12.5 (Agilent Technologies, Santa Clara, CA, USA).

### Chromatin immunoprecipitation

HUVECs were treated with 10 μM of pemafibrate for 24 h and crosslinked with 1% formaldehyde for 10 min at room temperature. After neutralization by the addition of 0.2 M glycine, cells were harvested, resuspended in lysis buffer (13.6 mM Tris–HCl, 166.7 mM Nacl, 0.8% SDS, 1.2 mM EDTA, 0.9% Triton X-100; pH 8.0). Chromatin DNA was sonicated with Sonifier 250 (Branson, Danbury, CT, USA) (output 4, duty cycle 60%, 20 s × 6 times) to generate approximately 0.5-kb fragments. Chromatin immunoprecipitation (ChIP) was performed using the antibodies listed in Supplementary Table S1 prebound to Dynabeads Protein G (Life Technologies/Thermo Fisher Scientific, South San Francisco, CA, USA). ChIP DNA was purified with the QIAquick PCR Purification Kit (Qiagen, Germantown, MD, USA) and quantified using a Qubit Fluorometer and dsDNA HS assay Kit (Life Technologies/Thermo Fisher Scientific). ChIP sequencing (ChIP-seq) was performed with an Illumina/Solexa sequencer as previously described^20–28,49–51^.

### ChIP-qPCR analysis

ChIP samples were analyzed by quantitative PCR using the gene-specific primers listed in Supplementary Table S2. ChIP signals were divided by no-antibody signals (input DNA) and presented as fold enrichment.

### Quantitative RT-PCR

Total RNA was isolated using Isogen (Fujifilm Wako Chemicals) according to the manufacturer’s protocol^49^. cDNA was synthesized from 1 μg of total RNA using oligo-dT primers and SuperScript II reverse transcriptase (Life Technologies). Quantitative PCR was performed using an ABI PRISM 7900HT sequence detection system (Applied Biosystems, Foster City, CA, USA). *PPIB* or *ACTB* was used as an invariant control. A list of primers for quantitative RT-PCR used in this study is shown in Supplementary Table S3.

### Immunoblot analysis

Protein samples obtained from HUVECs, HRMECs, or rat retinas were separated on 10% SDS-PAGE and electrophoretically transferred onto nitrocellulose membranes. The membranes were blocked with 5% skim milk in PBS containing 0.1% (v/v) Tween-20 for 1 h and incubated with primary antibody at 4 °C for 24 h. After washing, the membranes were incubated with secondary antibody conjugated with horseradish peroxidase at room temperature for 1 h. Immunoblots were visualized by chemiluminescence using Super Signal West Dura Extended Duration Substrate (Thermo Fisher Scientific), and luminescent images were analyzed with an ImageQuant LAS 4000mini (GE Healthcare, Piscataway, NJ, USA). A list of primary antibodies used in this study is shown in Supplementary Table S1.

### Luciferase reporter assay

The luciferase reporter assay was performed with the Beta-Glo assay system (Promega, Madison, WI, USA). HUVECs seeded on 24-well plates at 50% confluence in Opti-MEM (Life Technologies/Thermo Fisher Scientific) were transfected with 0.2 µg of reporter plasmid. HUVECs were incubated for 24 h in normal growth medium and then treated with 100 μM of fenofibric acid or vehicle (DMSO). Luciferase assays were performed 48 h after fenofibric acid treatment. Luciferase activity was normalized to the β-gal activity level.

### Type 1 diabetes rat model

Type 1 diabetes was induced in male Wistar rats by an intraperitoneal injection of STZ (60 mg/kg). Briefly, 6 to 8 weeks after STZ injection, the rats were fed pemafibrate (10 mg/kg) for 2 weeks. Intravitreal injection of control or *Thbd* siRNA was performed 6 and 7 weeks after STZ injections. The rats were euthanized 8 weeks after STZ injection. Rats in which blood glucose levels were greater than 300 mg/dl at tissue harvesting were defined as diabetic and used for the experiments.

### Retinal vascular leukostasis ssay

The retinal vascular leukostasis assay was performed as described previously^38^. Briefly, rats were deeply anesthetized, and PBS was injected into the left ventricle. The anesthetized rats were perfused with PBS to remove nonadherent leukocytes in vessels. After injection of PBS, FITC-conjugated concanavalin-A (40 μg/ml) (Vector Laboratories, Burlingame, CA, USA) was injected into the left ventricle. The retinas were surgically isolated and then flat mounted. FITC-labeled adherent leukocytes in the vasculature were counted under a fluorescence microscope by an operator masked to treatment allocation.

### Measurement of vascular leakage in the retina

Vascular leakage in the rat retinas was investigated using fluorescein angiography, as previously described^52^. Briefly, rats were deeply anesthetized, and FITC-dextran (Sigma-Aldrich, St. Louis, MO, USA) was injected into the left ventricle. After 5 min, the retinas were flat mounted and observed under a fluorescence microscope. Vascular leakage was quantitatively analyzed using Image J software by determining florescence intensities of FITC-dextran in the retina vessels.

### Study approval

All animal protocols in this study were performed in accordance with the institutional guidelines and were approved by the St. Marianna University Graduate School of Medicine Institutional Animal Care and Use Committee.

## Supplementary information

Supplementary file1 (DOCX 3252 kb)

## Data Availability

All data generated or analyzed during this study are included in the published article (and its supplementary files).
