# Supplementary material for: PPARα activation directly upregulates thrombomodulin in the diabetic retina
Source: Sci Rep. 2020 Jul 2;10:10837. doi: 10.1038/s41598-020-67579-1 (PMC7331602; doi:10.1038/s41598-020-67579-1)
Supplement: Supplementary file 1 — Supplementary file1 (DOCX 3252 kb) [file 41598_2020_67579_MOESM1_ESM.docx]

**PPARα activation directly upregulates thrombomodulin in the diabetic retina**

**Authors and affiliations**

Akira Shiono,^1^ Hiroki Sasaki,^1^ Reio Sekine,^1^ Yohei Abe,^2^ Yoshihiro Matsumura,^2^ Takeshi Inagaki,^3^ Toshiya Tanaka,^4^ Tatsuhiko Kodama,^4^ Hiroyuki Aburatani,^5^Juro Sakai,^2,6^ Hitoshi Takagi^1^

^1^Department of Ophthalmology, St. Marianna University of Medicine, 2-16-1 Sugao, Miyamae-ku, Kawasaki, Kanagawa, Japan; ^2^Division of Metabolic Medicine, The University of Tokyo, RCAST, 4-6-1 Komaba, Meguro-ku, Tokyo, Japan; ^3^Laboratory of Epigenetics and Metabolism, IMCR, Gunma University, 3-39-15 Showa-cho, Maebashi, Gunma, Japan; ^4^Research Center for Advanced Science and Technology, The University of Tokyo, 4-6-1 Komaba, Meguro-ku, Tokyo, Japan; ^5^Genome Science Division, The University of Tokyo, 4-6-1 Komaba, Meguro-ku, Tokyo, Japan; ^6^Molecular Physiology and Metabolism Division ,Tohoku University Graduate school of Medicine,2-1, Seiryo-cho, Aoba, Sendai, Miyagi, Japan

Corresponding author: Hitoshi Takagi

M.D., Ph.D,

Professor of Department of Ophthalmology, St. Marianna University of Medicine

2-16-1 Sugao, Miyamae-ku, Kawasaki, Kanagawa, Japan

Tel: +81-44-977-8111

Fax: +81-044-976-7435

E-mail: htakagimarianna@gmail.com

Supplementary Table S1. Antibodies used in this study.

| Antibody |  | Source | Catalog no./clone no.　or/and RRID | Dilution or concentration |
| --- | --- | --- | --- | --- |
| Anti-PPARα | Monoclonal | Our laboratory | IgG-H0723 | 10 µg ml^–1^ for ChIP |
| Anti-RXRα | Monoclonal | Our laboratory | IgG-1920 | 10 µg ml^–1^ for ChIP |
| Anti-thrombomodulin | Polyclonal | Life Span BioSciences | LS-C352932 | 1:500 for IB |
| Anti-CCL2 | Monoclonal | Millipore | MABN712 | 1:500 for IB |
| Anti-VCAM1 | Monoclonal | abcam | Ab134047  AB_2721053 | 1:1000 for IB |
| Anti-ICAM | Polyclonal | R&D Systems | AF583  AB_416687 | 1:1000 for IB |
| Anti-β-actin | Monoclonal | Sigma-Aldrich | A2228  AB_476697 | 1:5000 for IB |

Supplementary Table S2. ChIP-qPCR primers used in this study

| Gene | Sequence | | Amplified region |
| --- | --- | --- | --- |
|  | Forward primer | Reverse primer |  |
| *THBD* | 5'-AAAGGAAGGAAGTGCCTGGT-3' | 5'-AGGGCAGGGTTTACTCATCC-3' | THBD  (–0.1 kb) |
| *PPIB* | 5'-GTGGCTCAGGTT GTCCCTAC-3' | 5'-CCCTAAGCACTCGCC TACTG-3' | Cyclo  (gene body) |

Supplementary Table S3. RT-qPCR primers used in this study

| Gene | Sequence | |
| --- | --- | --- |
|  | Forward primer | Reverse primer |
| Human *PDK4* | 5-′GAGGTGGTGTTCCCCTGAGAATT-3' | 5′-CAAAACCAGCCAAAGGAGCATT-3' |
| Human *THBD* | 5'-AGCAAGCCCCACTTATTCCC-3' | 5'-GGGTGACTCAGGTGAGTTGG-3' |
| Human *PPIB* | 5'-TGGTTCCCAGTTTTTCATCTGC-3' | 5'-CCATGGCCTCCACAATATTCA-3' |
| Rat *Pdk4* | 5'-ATGCCCCTTTGGCTGGTTTT-3' | 5'-GGCATCTGTCCCATAGCCTG-3' |
| Rat *Thbd* | 5'-GATCTCCATTGCCAGCCT-3' | 5'-CACGTGCTGCAGTACTACCT-3' |
| Rat *Actb* | 5'-AACACCCCAGCCATGTACG-3' | 5'-ATGTCACGCACGATTTCCC-3' |

Supplementary Figure S1. Original Blots for Figure2.

Figure S1a. Full unedited gel for Figure2.





Figure S1b. Figure 2 HUVEC and HRMEC THBD lane



 Figure S1c. Figure 2 HUVEC and HRMEC THBD



 Figure S1d. Figure 2 HUVEC and HRMEC bactin lane



 Figure S1e.Figure 2 HUVEC and HRMEC bactine


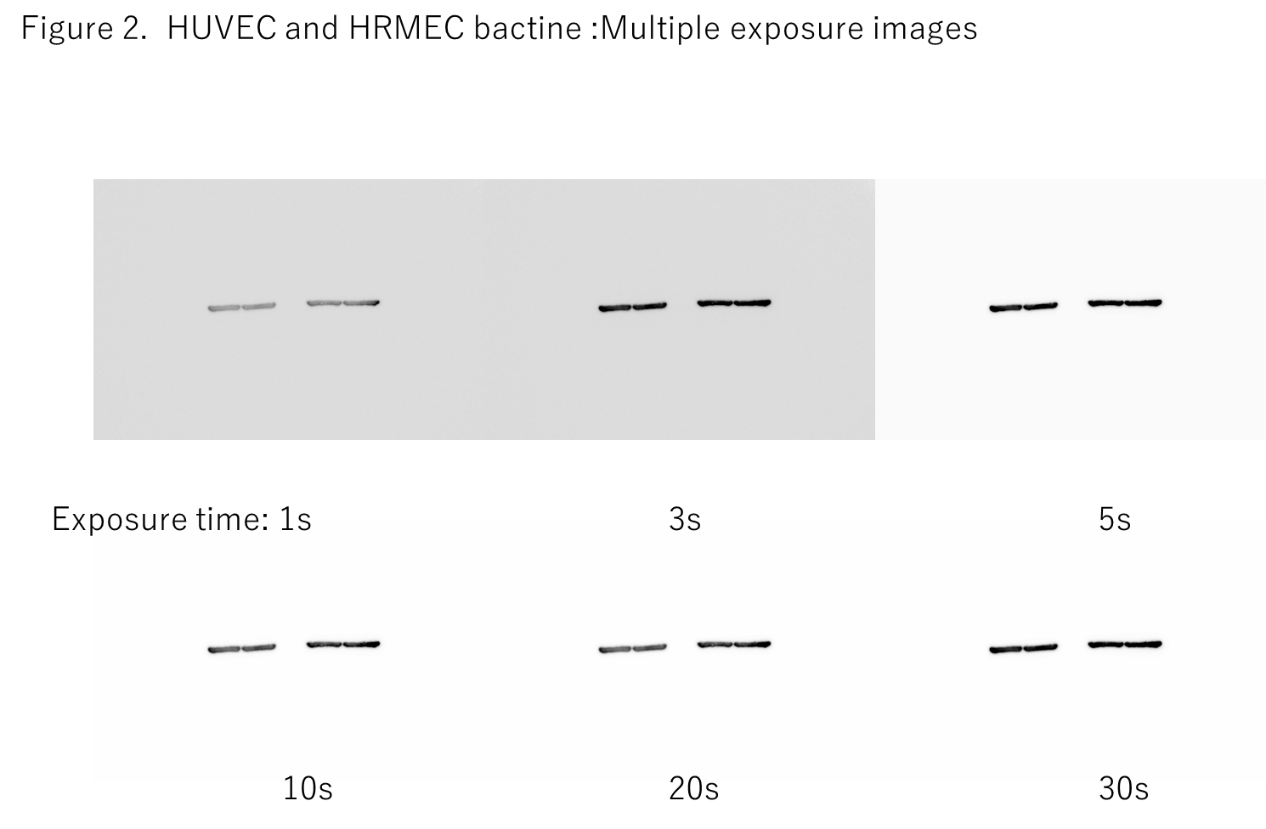


Figure S1f. HUVEC and HRMEC bactine

Supplementary Figure S2. Original Blots for Figure 3.

Figure S2a. Full unedited gel for Figure 3c.





Figure S2b. Figure 3c THBD siRNA lane



 Figure S2c. Figure 3c THBD siRNA



 Figure S2d. Figure 3c bactine lane





Figure S2e. Figure 3c bactine

Figure S2f . Full unedited gel for Figure 3d.





Figure S2g. Figure 3d ICAM lane





Figure S2h. Figure 3d ICAM





Figure S2i.Figure 3d MCP1 lane





Figure S2j.Figure 3d MCP1





Figure S2k.Figure 3d VCAM-1 lane





Figure S2l.Figure 3d VCAM-1





Figure S2m.Figure 3d bactin
